# Prediction of hypotension events with physiologic vital sign signatures in the intensive care unit

**DOI:** 10.1186/s13054-020-03379-3

**Published:** 2020-11-25

**Authors:** Joo Heung Yoon, Vincent Jeanselme, Artur Dubrawski, Marilyn Hravnak, Michael R. Pinsky, Gilles Clermont

**Affiliations:** 1 Division of Pulmonary, Allergy, and Critical Care Medicine, School of Medicine, University of Pittsburgh, 200 Lothrop street, Pittsburgh, PA 15213 USA; 2 Department of Critical Care Medicine, School of Medicine, University of Pittsburgh, Pittsburgh, PA USA; 3 Auton Lab, School of Computer Science, Carnegie Mellon University, Pittsburgh, PA USA; 4 School of Nursing, University of Pittsburgh, Pittsburgh, PA USA

**Keywords:** Machine learning, Artificial intelligence, Hypotension, Prediction

## Abstract

**Background:**

Even brief hypotension is associated with increased morbidity and mortality. We developed a machine learning model to predict the initial hypotension event among intensive care unit (ICU) patients and designed an alert system for bedside implementation.

**Materials and methods:**

From the Medical Information Mart for Intensive Care III (MIMIC-3) dataset, minute-by-minute vital signs were extracted. A hypotension event was defined as at least five measurements within a 10-min period of systolic blood pressure ≤ 90 mmHg and mean arterial pressure ≤ 60 mmHg. Using time series data from 30-min overlapping time windows, a random forest (RF) classifier was used to predict risk of hypotension every minute. Chronologically, the first half of extracted data was used to train the model, and the second half was used to validate the trained model. The model’s performance was measured with area under the receiver operating characteristic curve (AUROC) and area under the precision recall curve (AUPRC). Hypotension alerts were generated using risk score time series, a stacked RF model. A lockout time were applied for real-life implementation.

**Results:**

We identified 1307 subjects (1580 ICU stays) as the hypotension group and 1619 subjects (2279 ICU stays) as the non-hypotension group. The RF model showed AUROC of 0.93 and 0.88 at 15 and 60 min, respectively, before hypotension, and AUPRC of 0.77 at 60 min before. Risk score trajectories revealed 80% and > 60% of hypotension predicted at 15 and 60 min before the hypotension, respectively. The stacked model with 15-min lockout produced on average 0.79 alerts/subject/hour (sensitivity 92.4%).

**Conclusion:**

Clinically significant hypotension events in the ICU can be predicted at least 1 h before the initial hypotension episode. With a highly sensitive and reliable practical alert system, a vast majority of future hypotension could be captured, suggesting potential real-life utility.

## Introduction

Hypotension is known to be the most consistent manifestation of decompensated shock leading to major organ failure and death [1]. Hypotension, along with other chronic risk factors, is associated with an increased chance of acute kidney injury, myocardial ischemia, and mortality [2, 3]. However, the underlying signatures from hemodynamic monitoring variables that portend impending hypotension are not clearly identified [4]. Identifying that a patient is on a trajectory to a hypotensive episode with sufficient lead time could lead to effective mitigation of hypotension and possibly improved outcomes. Moreover, current treatment protocols for hypotension may themselves be associated with unwanted consequences such as excessive resuscitation and worsening of acute lung injury [5, 6].

Several early warning scores have been introduced to identify patients at risk for decompensation and trigger escalation of care [7–9]. However, most are manually calculated and often require additional data to be entered beyond what is readily available from monitors or electronic health records, limiting their utility [10]. More importantly, current metrics are unable to provide reliable, continuous feedback to clinicians who need to make time-sensitive decisions for rapidly fluctuating conditions. Even recent publications on data-driven prediction models lack clinically applicable implementation strategies [11–13]. Therefore, the creation of a real-time, continuous, translationally relevant forecasting system, which goes beyond a simple prediction model to include an additional enrichment layer to enhance alerting reliability, would favor successful implementation at the bedside by enhancing reliability and reducing false alarms.

With parsimonious use of multi-granular features and application of machine learning (ML) algorithms, we previously demonstrated the value of an early warning system to predict cardiorespiratory insufficiency (CRI) with high accuracy in step-down units [14] as well as tachycardia prediction in the ICU [15]. We have also demonstrated that the risk of CRI evolves along heterogeneous but repeatable trajectories, enabling early forecasting of the onset of crisis [16]. We hypothesized that clinically significant hypotension, a frequent form of CRI, could also be predicted and that this prediction could be actioned into a practical alert system for critically ill patients.

## Materials and methods

### Study population

A publicly available retrospective multigranular dataset, the Medical Information Mart for Intensive Care III (MIMIC-3), collected between 2000 and 2014 from a tertiary care hospital in Boston, MA, was used as data source [17]. Subjects with age ≥ 18 that have all vital signs and clinical records were selected. Algorithms were applied to identify hypotension events, as described below, to classify the source population into ‘hypotension group’ (subjects experiencing at least one hypotension event) and ‘non-hypotension group’ (subjects with no hypotension events). To enhance specificity of identification of the first recorded event of hypotension, we further excluded from the hypotension group subjects who had received vasopressors, any amount of crystalloid bolus, or packed red blood cell transfusion two hours prior to the subject’s initial (first) hypotension event. Only the first hypotension event was targeted for prediction. Subjects admitted to the ICU prior to the median date of hospital admission were used for model selection and training, while those admitted later than the median admission date were used for validation.

### Defining hypotension, preprocessing of data, and feature engineering

To identify clinically relevant hypotension, the following steps were taken. First, the threshold for hypotension was determined as systolic blood pressure (SBP) ≤ 90 mmHg and mean arterial pressure (MAP) ≤ 60 mmHg [18]. Second, at least 5 of 10 consecutive blood pressure readings (5 out of 10 min, with discrete data points) had to be below the thresholds. Third, if there was a gap of 2 min or less between two periods under the thresholds, these two periods were combined into a single hypotension event (Additional file 1: Figure S1).

In preprocessing, physiologically implausible values were removed including SBP, diastolic blood pressure (DBP), or MAP < 10 or > 400 mmHg; respiratory rate < 1 or > 100/min, heart rate < 10 or > 400/min, SpO_2_ < 10%. Missing values were imputed using a moving average of the three previous values assuming signal stability during that missing data time period if less than 10 min. Interpolation with the use of future data was avoided. The performance of this imputation method was examined by comparing statistics of imputed data windows to ground truth provided by data segments without missing data.

**Fig. 1:**
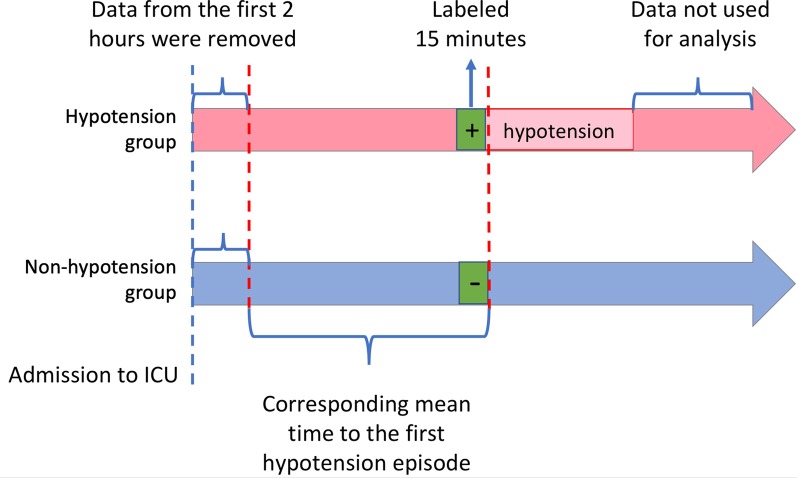
Schematic illustration to assess the performance of the hypotension prediction model on a finite time horizon (2 h). From the developmental cohort, the last 5 min before hypotension episode for the hypotension group was labeled and trained as positive, and 15-min long data before the last 2 h and 15 min were labeled and trained as negative

We computed features from raw vital signs time series including statistical values (variance, quartiles, mean, median, min, max), frequency domain features using discrete Fourier transform [19], as well as exponentially weighted moving average (EWMA, moving average with increased weighing for recent events) [20]. Features were computed on windows of different durations (5, 10, 30, and 60 min), rolling every minute. To appropriately label true hypotension events, we assumed all windows from hypotension subject in the training set had a positive label (hypotension) for the last 15 min prior to the hypotension event. For the non-hypotension group, we assigned negative labels to non-hypotension subject windows selected such that their ‘onset’ time was defined as within 15 min of the mean time on the first hypotension onset from ICU admission in the hypotension subjects (Fig. 1). Matching similar time lapse in the non-hypotensive group with the time to the first hypotension event in hypotension group was performed to generate the negative label with minimal potential bias, as the overall trajectories during the first few hours, or the last few hours of ICU stay could introduce greater non-physiologic confounders. The decision of using a 15-min timeframe was made considering practical clinical response time in anticipation of a hypotensive event and also objectively supported by a t-stochastic neighbor embedding (t-SNE) analysis [21], an unsupervised ML model that estimates the divergent time point of the two groups before hypotension (Additional file 2: Figure S2).

### Model training and validation

We used a random forest (RF) classifier, K-nearest neighbor (KNN) classifier, gradient boosted trees, and logistic regression with L2 regularization models [22, 23] with tenfold cross-validation process on the training set (development cohort) (Additional file 3: Figure S3). The best performing model in the training set was applied to the validation set. Performances of those different supervised ML models were compared using the area under the receiver operating characteristic curve (AUROC) and its evolution over time. Since the number of the hypotension group and the non-hypotension group subjects were unequal (unbalanced), we also computed area under the precision recall curves (AUPRC), according to recent recommendations following the transparent reporting of a multivariable prediction model for individual prognosis or diagnosis (TRIPOD) statement [24, 25]. Model calibration was evaluated using the Brier’s score [26, 27]. A brief explanation on models used, training methods, and performance evaluation techniques can be found in supplementary glossary (Additional File 4: Glossary).

### Hypotension prediction risk score trajectories

A risk score (a number between 0 and 1, representing the relative probability of future hypotension at the end of the observation window) was generated every minute for each subject in the validation cohort, generating individualized risk trajectories for the entire ICU stay of a subject. Due to missing data, not all subjects had risk scores computed every minute before hypotension or the end of the monitoring window.

### Operational performance of the hypotension alert system

To better understand how a hypotension forecasting model would perform prospectively when deployed as an alerting system, we identified exceedances per hour per subject, of the predicted hypotension risk score beyond various risk score thresholds. To further determine whether an exceedance of the risk score qualifies as a system alert, we employed a stacked RF model. This two-step (stacked) model (Additional file 5: Figure S4) was first trained with tenfold cross-validation, then underwent out-of-sample subjects validation using additional features including time since admission, the average, minimum, maximum and standard deviation of risk scores created by the first model, over the last 5, 10, and 30 min prior to the current time on a moving window. Lastly, a lockout period of 15 min (alert will not be generated if it were to occur within 15 min after the previous alert) was used to prevent an excessive number of alerts, either true or false. The alert-level performance was evaluated with true positive alert rate and total alerts per subject per hour, to evaluate whether alerts received could be trusted, and how many alerts clinicians would receive at the bedside. The subject(patient)-level performance was assessed with the probability of a future hypotension event following an alert (positive predictive value, PPV) and recall rate (1—sensitivity) to demonstrate how likely an alert system would predict, and fail to predict hypotension events, respectively.

## Results

**Fig. 2:**
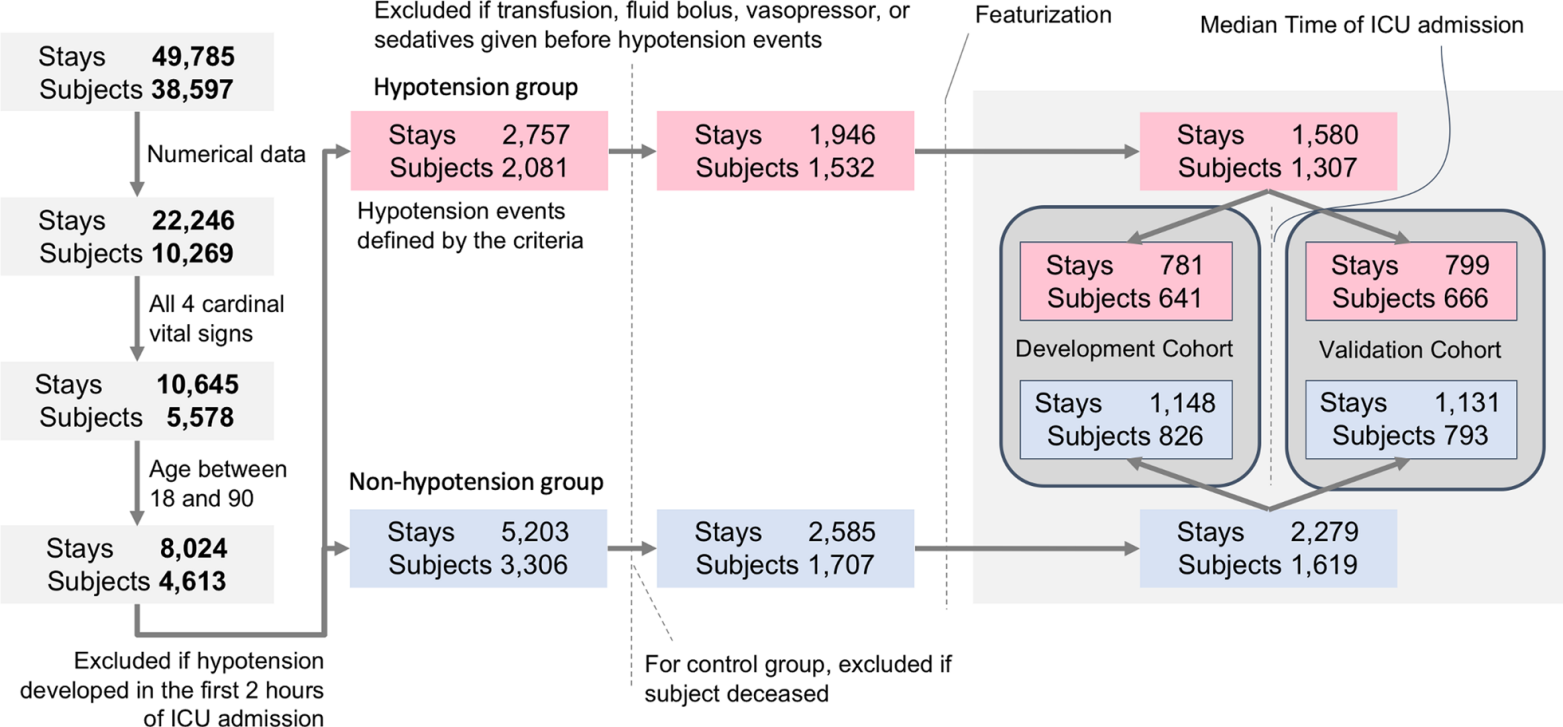
Data extraction pipeline. From the initial MIMIC3 database, inclusion and exclusion criteria were applied, along with the definition of hypotension event. Then, feature selection was performed to derive the hypotension (subjects experienced hypotension events during the ICU stay) and the non-hypotension (those without the hypotension event) groups

From a source population of 10,269 subjects with 22,246 ICU stays, we identified 1532 subjects (1946 ICU stays) as the hypotension group, and 1707 subjects (2585 ICU stays) as the non-hypotension group (Fig. 2). The development cohort included 641 hypotension subjects (781 ICU stays) and 826 non-hypotension subjects (1148 ICU stays). The validation cohort includes 666 hypotension subjects (799 ICU stays) and 793 non-hypotension subjects (1131 ICU stays). The development and validation cohorts were similar in size, gender, types of ICU (i.e., Medical ICU, Cardiothoracic ICU, or Surgical ICU), and in-hospital mortality. Notably, age and length of hospital stay were slightly shorter in the developmental cohort (Additional file 6: Table S1). In comparing hypotension group and non-hypotension group, the hypotension group subjects were older (age 66.4 years vs. 60.8 years) and had longer hospital stays (11 days vs. 5.7 days). Otherwise both the hypotension and non-hypotension groups were similar in distribution of sex and ICU types (Additional file 7: Table S2). The mean time to the first hypotension event was 102 h and 16 min (standard deviation: 164 h and 23 min). The median time was 34 h and 28 min, with the interquartile range was 107 h and 45 min.

### Performance of machine learning algorithms

**Fig. 3:**
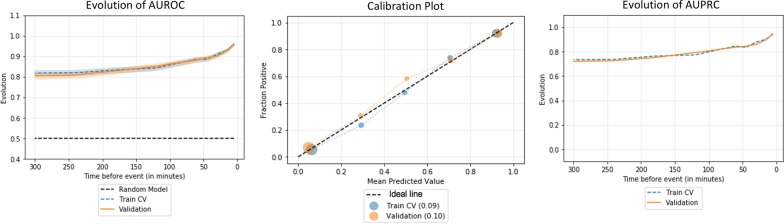
Performance evaluation for various supervised machine learning algorithms, with the evolution of area under the receiver operating characteristics (AUROC) over time (left), calibration plot with the Brier’s scores (center), and the evolution of the area under the precision–recall curve (AUPRC) over time (right)

In selecting the best risk model, RF showed equivalent performances as visualized on the AUROC on the validation cohort, compared with KNN, logistic regression, and gradient boosted forest models. AUROC during the last 15 min and 1 h prior to the hypotension event were 0.93 and 0.88, respectively, on the validation cohort (Additional file 8: Figure S5, Left), with a good calibration score (Brier score 0.09) (Additional file 8: Figure S5, Center), with AUPRC of 0.90 and 0.83 (Additional file 8: Figure S5, Right). Given equivalence of predictive performance, but prevailing calibration score, we chose the RF algorithm to build a prediction model. Then, the performance of the RF model was verified with the validation cohort, with AUROC, calibration plot, and AUPRC (Fig. 3).

### Trajectory analysis

**Fig. 4:**
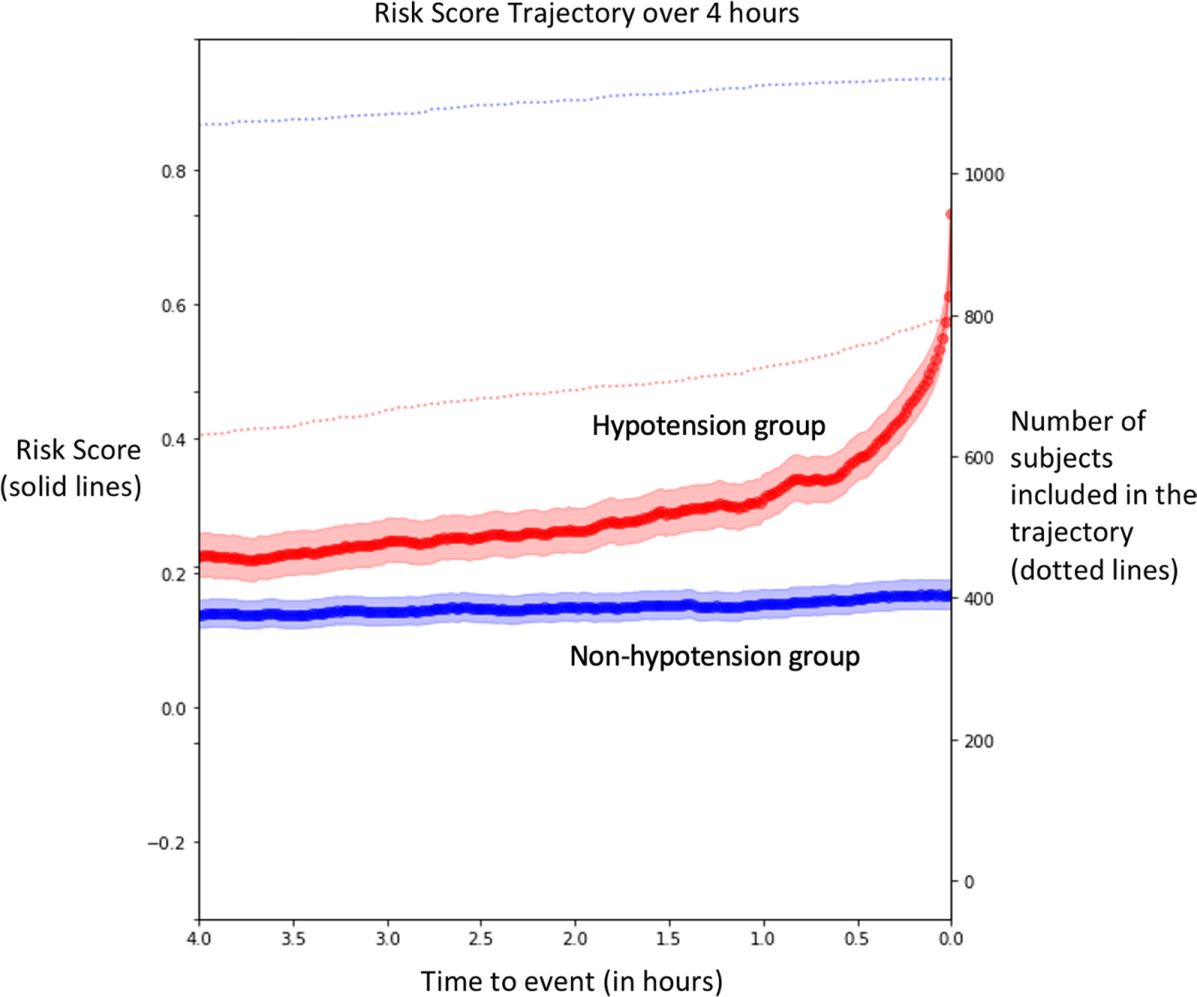
Average evolution of the risk scores for hypotension (red) and non-hypotension (blue) groups is projected as trajectories, extending to 4 h preceding hypotension events. Shaded areas represent 95% confidence intervals. Dotted lines indicate the number of hypotension and non-hypotension subjects used to derive the risk score trajectory points at a given horizon

With the missing values due to incomplete data, we had 793 hypotension subjects and 1131 non-hypotension subjects with risk score trajectories. The trajectories (Fig. 4) drawn with the validation cohort exhibited a clear separation in mean risk between hypotension and non-hypotension subjects from 3 h prior to hypotension. The separation became wider as the hypotension subjects are getting closer to the hypotension event, and the risk of hypotension subjects escalated rapidly from approximately 30 min prior to hypotension.

### Alert identification and operational usefulness

**Fig. 5:**
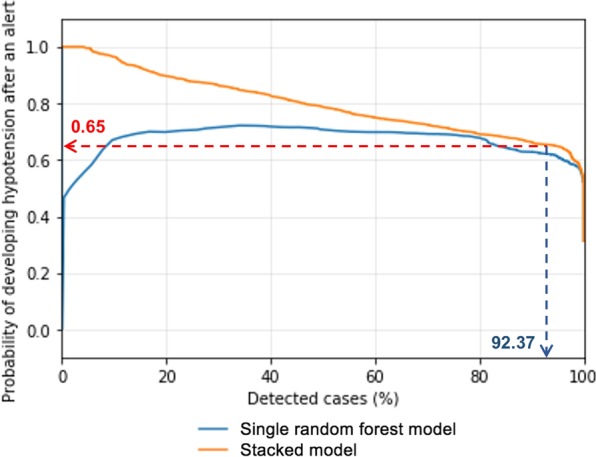
Relationship between the detected hypotension subjects (%) and the probability of hypotension after an alert between the stacked model (orange line) and single random forest model (blue line). Detected cases indicate the percentage of hypotension subjects our model successfully predicted as an alert before hypotension. At the risk score threshold of 0.5, the probability of hypotension in the future (positive predictive value) was approximately 0.65 (red dashed arrow), with 92.37% of hypotension events were captured (vertical blue dashed arrow)

We investigated the relationship between different risk score thresholds and the probability of future hypotension (alert). We first chose a threshold of 0.5, aiming to detect at least 90% of actual future hypotension events, which yielded 7.39 alerts/subject/hour on average, with a PPV of 57.7%. Using the stacked RF model, average alert frequency was reduced to 4.93 alerts/subject/hour at the same thresholds, with PPV improved to 65.2%. Adding a 15-min lockout period on the output of the stacked model further decreased alerts to 0.79 alerts/subject/hour. That means, with the lockout period, the probability of future hypotension with a single alert (PPV) will not be negatively impacted, but a clinician could expect much less alerts (every 75 min on average). Using the same thresholds, our model will have sensitivity of 92.4% (failing to predict hypotension for 7.6% of actual hypotension subjects) (Fig. 5). On the patient(subject)-level, the whole ICU data of a given subject showed the AUPRC of 0.68. When a random 1-h data from the hypotension and non-hypotension groups were used, the AUPRC was 0.91 in the validation cohort.

## Discussion

This study is one of the few to demonstrate the capacity of data-driven prediction of clinically significant hypotension events using a supervised ML algorithm to stream bedside monitoring vital sign data and is unique in providing an actionable roadmap for the design of an alerting system using a predictive model developed on retrospective data that has clinical utility. In this study, we focused on designing a ‘data-driven’ risk prediction system that has operational usefulness. Our approach is notable for a modeling component where we assess model performance using multiple methods and express dynamic changes in risk, and an implementation part where we design a two-step pipeline to increase the overall reliability of alerts and decrease alarm fatigue.

There are few studies using ML-based approaches to predict hypotension. A recent report describes the intraoperative prediction of hypotension 10–15 min earlier, with using data from a specialized commercially available noninvasive continuous arterial waveform sensor [11]. Our research demonstrated that this prediction task could be extended to ICU patients, with using relatively sparse (minute-by-minute) data, resulting in a longer prediction horizon (AUROC of 0.88 at 60 min prior to the event), linked with clinically relevant alerts (an average of 0.79 alerts/subject/hour, with a 15-min lockout time). Our strategy has practical benefits in implementation to secure time to prepare action plans and can be useful in a relatively resource-limited environment where continuous care by trained critical care providers is not available for immediate action.

Performance of the prediction model needs to be assessed with multiple methods. The previous study on the MIMIC II database has used vital signs and medication data to predict hypotension at 1-h prior to the event showed an AUROC of 0.934 [12]. Despite seemingly high AUROC, however, the study reported many negative samples (low pretest probability) and a deceptively low PPV of 0.151, limiting real-life feasibility of the resulting model. To bolster potential feasibility in implementation, we performed a multi-faceted performance evaluation by employing not only AUROC trajectory, but also AUPRC and calibration assessed using Brier’s score. Methodologically, model analysis relying only on the cumulative assessment of ROC curves could mislead the interpretation of such patterns in vital sign physiology, as AUROC itself does not address misclassification cost [28]. The addition of PRC allowed assessing the ability of the models to identify true positives when the groups are imbalanced, providing the full picture of the capacity of different models. Calibration study helped selecting the model type whose predictions are best aligned with posterior probability distributions.

Building risk score trajectories provides conceptual and practical advantages. Instantaneous scores may be subject to stochastic variations, erroneous entries, and artifacts, while trajectories provide historical context to a risk, which translate to the clinical concept of worsening or improving health state, perhaps in response to an intervention [29–31]. Second, studies suggest that the prognosis of critically ill patients is associated with early recognition and timely intervention of abrupt changes [32]. Early identification of dynamic risk changes on critically ill patients can be highly informative, as sudden unexpected physiologic deteriorations are common in the ICU (e.g., septic patients develop gastrointestinal bleeding with stress ulcer; or renal failure patients exhibit arrhythmias with severe hyperkalemia). In our study, prediction visualized with the risk score trajectory allowed early differentiation in the mean risk scores from at least 3 h prior to hypotension events, which objective metric does not rely on practitioners’ level of skill in interpretation. While this finding illustrates the power of our model, it needs to be interpreted with caution: The mean risk is never applicable to an individual patient; thus, some probability of an individual being outside of a common band should be provided with high interpersonal variances of risk scores in real life.

Prediction followed by a reliable alerting strategy of any critical event in real life would be the one of the holy grails of the ICU care, because of a highly variable and heterogeneous individual clinical pictures. A recent study confirmed this, as various cardiovascular states, reserves, or responses were observed when a standardized resuscitation protocol was employed during septic shock [33]. Delivering the prediction to the bedside is also challenging, as the alert should be actionable and linked to a meaningful management strategy. A recent study developed an intraoperative hypotension prediction index with an AUROC of 0.88 at 15 min, with excellent PPV and NPV [34], and was linked with an action plan in the operating room to treat predicted hypotension, resulted in less hypotension and less post-operative complications [35]. In our study, we conceptualized a predictive alert system performing well in the ICU environment where less frequent monitoring takes place than the operating room, along with a lockout design to minimize alarm fatigue. First, our two-step model demonstrated the utility of potential implementation with further increased true positive and decreased false positive rates. Then, a 15-min lockout period allowed the model to be more actionable by reducing the unwanted impact on alarm fatigue. Mitigating alarm fatigue is important, because it could be associated with failure to rescue, if clinicians ignore excessively frequent alarms even though they may carry critical information [36]. The 15-min lockout period was chosen to decrease repetitive alerts with similar clinical meaning, assuming that alerts more frequent than 15 min would not alter the rationale of management strategy. In a hypothetical 20-bed ICU, our model would alarm 16 times per hour, while without the lockout period, it would yield 159 alerts (7.93 × 20) per hour. A good example for this application would be a recent study used a rule-based model for cardiothoracic ICU patients to decrease alerts by 55% with lockout, while capturing almost all true clinical deterioration events [37]. Finally, with the alert-level and subject-level analyses of the alert system, we showed that a vast majority of future hypotension could be captured with a high sensitivity and an accountable probability, suggesting potential real-life utility.

Our work has several limitations. First, an external validation cohort using multicenter data was not used to confirm the performance of our model. Instead, we used an a priori separated out-of-sample validation set. We are currently collecting a large-scale multigranular ICU data in our institution and plan for further external cohort validation. Second, our operational definition of hypotension was based on conventional cutoff values, not specifically designed to meet the characteristics of the individual subjects. In addition, despite our preprocessing to minimize non-physiologic artifacts, there could still be artifacts fell within physiologic normal range and might have interfered the analysis. However, we postulated the effects of the remaining artifacts are likely minimally associated if the distribution of artifacts across vital sign samples are not systematic (i.e., random). Third, we developed a risk model for the first hypotension event in the ICU, to simplify the prediction task. An alerting system that would apply across the span of an admission would have to go beyond a first episode of hypotension. An alerting system for all hypotension episodes would need to integrate a different risk model for subsequent episodes, and this model would likely use clinical interventions and number of prior episodes as additional risk factors. We are currently developing such a model. We also excluded subjects that had received fluid bolus or vasopressor treatment within 2 h of a first hypotension event. This modeling choice, justified by the notion that such interventions were possibly linked to unwitnessed hypotension (prior to data availability) and thus that the first hypotensive episode recorded episode of hypotension in this subpopulation, was more likely to be a subsequent episode. These choices narrowed the potential utility of our alert system if deployed. Although our proposed alerted system could be tested in these circumstances, we would expect a decrease in performance. Fourth, we did not use temporal correlations between features imposed by physiological constraints as additional features. Expanding the set of variables to include more sophisticated and physiologically inspired features, and including higher granularity monitor data (e.g., waveforms) might achieve better algorithm performance. Lastly, our selection of hypotension events might have missed potential real events (false negatives), as acutely profound hypotension events are usually treated within minutes with fluid boluses resuscitation or vasopressor therapy. With a higher granularity dataset, we argue that individual trajectory and its triggering factors for future hypotension could be identified in a more sophisticated manner.

## Conclusions

Clinically relevant hypotension events can be predicted from minute-by-minute vital signs dataset with the use of machine learning approaches. This prediction can be integrated into a highly sensitive alert delivery system with low false alerts causing minimal alarm fatigue, with potential real-life utility.

## Supplementary information

**Additional file 1: Figure S1.** Illustration of definition for hypotension events, with duration, interval, and density criteria.

**Additional file 2: Figure S2.** Selection of optimal time window prior to hypotension event by using a t-stochastic neighbor embedding (t-SNE) embedding. Raw vital sign data were plotted from 1 h prior to hypotension event (or 1 h prior to the average time of hypotension event, in hypotension or non-hypotension groups). The t-SNE enforces data with similar vital signs to be projected closely. With this manifold representation of data, distribution of data points for the hypotension group was shown (Left). The colors of dots represented the time horizon towards the hypotension event. The data points of the non-hypotension group (dark blue dots) overlapped onto a major portion of the hypotension group, suggesting the non-hypotension group vital sign behaviors are similar to those of the hypotension group. However, when compared to the hypotension group only (Right), the overlapping does not involve certain areas, especially red-colored dots, representing hypotension subjects at approximately 15 min before the hypotensive event. This suggests that the vital sign patterns are probabilistically different between the two groups about 15 min prior to the event.

**Additional file 3: Figure S3.** Splitting the data into training and validation subset, according to the median time of ICU admission. The blue part (Left) was used to complete the hypotension prediction model with training followed by 10-fold cross validation methods. Then the pre-separated yellow part (Right) was used to validate the model afterwards.

**Additional file 4**: Glossary for machine learning models, model training methods, and model performance evaluation techniques used.

**Additional file 5: Figure S4.** A flowchart of stacked model (two-step approach) to derive alert scores. The first step model is used a random forest regressor trained on the input vital sign features to predict risk scores. The second step model is another random forest with expanded feature set to decrease false alerts. The additional features include time since admission, average, minimum, maximum, and standard deviation of risk scores obtained from the first model, over the last 5, 10, and 30 min prior to the current time. Both random forest models were trained and tested with tenfold cross validation.

**Additional file 6: Table S1.** Demographic and clinical characteristics of training and validation cohorts.

**Additional file 7: Table S2.** Demographic and clinical characteristics of the training and validation cohort for Hypotension group and non-hypotension group across all subjects.

**Additional file 8: Figure S5.** Performance evaluation for various supervised machine learning algorithms on the training cohort, with the evolution of area under the receiver operating characteristics (AUROC) over time (Left), calibration plot with the Brier’s score (Center), and the evolution of the area under the precision–recall curve (AUPRC) over time (Right). Note the random forest had similar performance to other methods in terms of AUROC and AUPRC distribution, but it demonstrated superior calibration metric.

## Data Availability

The vital sign data for this study (MIMIC-III) was published by Alistair E.W. Johnson et al. in 2016, and its reference is as hyperlinked: https://www.nature.com/articles/sdata201635. The actual data could be obtained through website as hyperlinked: https://mimic.physionet.org.

## References

[1] Fitch W, Mackenzie ET, Harper AM (1975). Effects of decreasing arterial blood pressure on cerebral blood flow in the baboon. Influence of the sympathetic nervous system. Circ Res.

[2] Walsh M, Devereaux PJ, Garg AX (2013). Relationship between intraoperative mean arterial pressure and clinical outcomes after noncardiac surgery: toward an empirical definition of hypotension. Anesthesiology.

[3] Ahuja S, Mascha EJ, Yang D (2020). Association of intraoperative radial arterial systolic, diastolic, mean, and pulse pressures with myocardial and acute kidney injury after noncardiac surgery: a retrospective cohort analysis. Anesthesiology.

[4] Arlati S, Brenna S, Prencipe L (2000). Myocardial necrosis in ICU patients with acute non-cardiac disease: a prospective study. Intensive Care Med.

[5] Kelm DJ, Perrin JT, Cartin-Ceba R (2015). Fluid overload in patients with severe sepsis and septic shock treated with early goal-directed therapy is associated with increased acute need for fluid-related medical interventions and hospital death. Shock.

[6] van Mourik N, Metske HA, Hofstra JJ (2019). Cumulative fluid balance predicts mortality and increases time on mechanical ventilation in ARDS patients: an observational cohort study. PLoS ONE.

[7] Knaus WA, Draper EA, Wagner DP, Zimmerman JE (1985). APACHE II: a severity of disease classification system. Crit Care Med.

[8] Le Gall JR, Lemeshow S, Saulnier F (1993). A new Simplified Acute Physiology Score (SAPS II) based on a European/North American multicenter study. JAMA.

[9] Vincent JL, Moreno R, Takala J, Willatts S, De Mendonca A, Bruining H, Reinhart CK, Suter PM, Thijs LG (1996). The SOFA (Sepsis-related Organ Failure Assessment) score to describe organ dysfunction/failure. On behalf of the Working Group on Sepsis-Related Problems of the European Society of Intensive Care Medicine. Intensive Care Med.

[10] Rothman M, Rothman S, Beals J (2013). Development and validation of a continuous measure of patient condition using the Electronic Medical Record. J Biomed Inform.

[11] Maheshwari K, Buddi S, Jian Z (2020). Performance of the Hypotension Prediction Index with non-invasive arterial pressure waveforms in non-cardiac surgical patients. J Clin Monit Comput.

[12] Lee J, Mark RG (2010). A hypotension episode predictor for intensive care based on heart rate and blood pressure time series. Comput Cardiol.

[13] Cherifa M, Blet A, Chambaz A, Gayat E, Resche-Rigon M, Pirracchio R (2020). Prediction of an acute hypotension episode during an ICU hospitalization with a super-learner machine learning algorithm. Anesth Analg.

[14] Hravnak M, Devita MA, Clontz A, Edwards L, Valenta C, Pinsky MR (2011). Cardiorespiratory instability before and after implementing an integrated monitoring system. Crit Care Med.

[15] Yoon JH, Mu L, Chen L, Dubrawski A, Hravnak M, Pinsky MR, Clermont G (2019). Predicting tachycardia as a surrogate for instability in the intensive care unit. J Clin Monit Comput.

[16] Chen L, Ogundele O, Clermont G, Hravnak M, Pinsky MR, Dubrawski AW (2017). Dynamic and personalized risk forecast in step-down units. Implications for monitoring paradigms. Ann Am Thorac Soc.

[17] Johnson AE, Pollard TJ, Shen L, Lehman LW, Feng M, Ghassemi M, Moody B, Szolovits P, Celi LA, Mark RG (2016). MIMIC-III, a freely accessible critical care database. Sci Data.

[18] Maheshwari K, Nathanson BH, Munson SH, Khangulov V, Stevens M, Badani H, Khanna AK, Sessler DI (2018). The relationship between ICU hypotension and in-hospital mortality and morbidity in septic patients. Intensive Care Med.

[19] Winograd S (1978). On Computing the discrete Fourier transform. Math Comput.

[20] Kenney JF, Keeping ES (1951) Mathematics of statistics, Part 2, 2nd edn. Van Nostrand, Princeton

[21] van der Maaten LJP, Hinton GE (2008). Visualizing high-dimensional data using t-SNE. Journal of Machine Learning Research.

[22] Clarke B, Fokoue E, Zhang HH (2009). Springer series in statistics—principles and theory for data mining and machine learning.

[23] Pedregosa G, Gramfort A, Michel V, Thirion B, Grisel O, Blondel M, Prettenhofer P, Weiss R, Dubourg V, Vanderplas J, Passos A, Cournapeau D, Brucher M, Perrot FV (2011). Scikit-learn: machine learning in Python. J Mach Learn Res.

[24] Moons KG, Altman DG, Reitsma JB (2015). Transparent Reporting of a multivariable prediction model for Individual Prognosis or Diagnosis (TRIPOD): explanation and elaboration. Ann Intern Med.

[25] Leisman DE, Harhay MO, Lederer DJ (2020). Development and reporting of prediction models: guidance for authors from editors of respiratory, sleep, and critical care journals. Crit Care Med.

[26] Rufibach K (2010). Use of Brier score to assess binary predictions. J Clin Epidemiol.

[27] Moons KGM, Wolff RF, Riley RD (2019). PROBAST: a tool to assess risk of bias and applicability of prediction model studies: explanation and elaboration. Ann Intern Med.

[28] Halligan S, Altman DG, Mallett S (2015). Disadvantages of using the area under the receiver operating characteristic curve to assess imaging tests: a discussion and proposal for an alternative approach. Eur Radiol.

[29] Yoon JH, Pinsky MR (2018). Predicting adverse hemodynamic events in critically ill patients. Curr Opin Crit Care.

[30] Lake DE, Fairchild KD, Moorman JR (2014). Complex signals bioinformatics: evaluation of heart rate characteristics monitoring as a novel risk marker for neonatal sepsis. J Clin Monit Comput.

[31] Churpek MM, Yuen TC, Winslow C (2014). Multicenter development and validation of a risk stratification tool for ward patients. Am J Respir Crit Care Med.

[32] Seymour CW, Gesten F, Prescott HC, Friedrich ME, Iwashyna TJ, Phillips GS, Lemeshow S, Osborn T, Terry KM, Levy MM (2017). Time to treatment and mortality during mandated emergency care for sepsis. N Engl J Med.

[33] Guarracino F, Bertini P, Pinsky MR (2019). Cardiovascular determinants of resuscitation from sepsis and septic shock. Crit Care.

[34] Davies SJ, Vistisen ST, Jian Z (2020). Ability of an arterial waveform analysis—derived hypotension prediction index to predict future hypotensive events in surgical patients. Anesth Analg.

[35] Wijnberge M, Geerts BF, Hol L (2020). Effect of a machine learning-derived early warning system for intraoperative hypotension vs standard care on depth and duration of intraoperative hypotension during elective noncardiac surgery. The HYPE Randomized Clinical Trial. JAMA.

[36] Sendelbach S, Funk M (2013). Alarm fatigue: a patient safety concern. AANC Adv Crit Care.

[37] King A, Fortino K, Stevens N (2012). Evaluation of a smart alarm for intensive care using clinical data. Conf Proc IEEE Eng Med Biol Soc.

