# Supplementary figures and images for: Prediction of hypotension events with physiologic vital sign signatures in the intensive care unit
Source: Crit Care. 2020 Nov 25;24:661. doi: 10.1186/s13054-020-03379-3 (PMC7687996; doi:10.1186/s13054-020-03379-3)

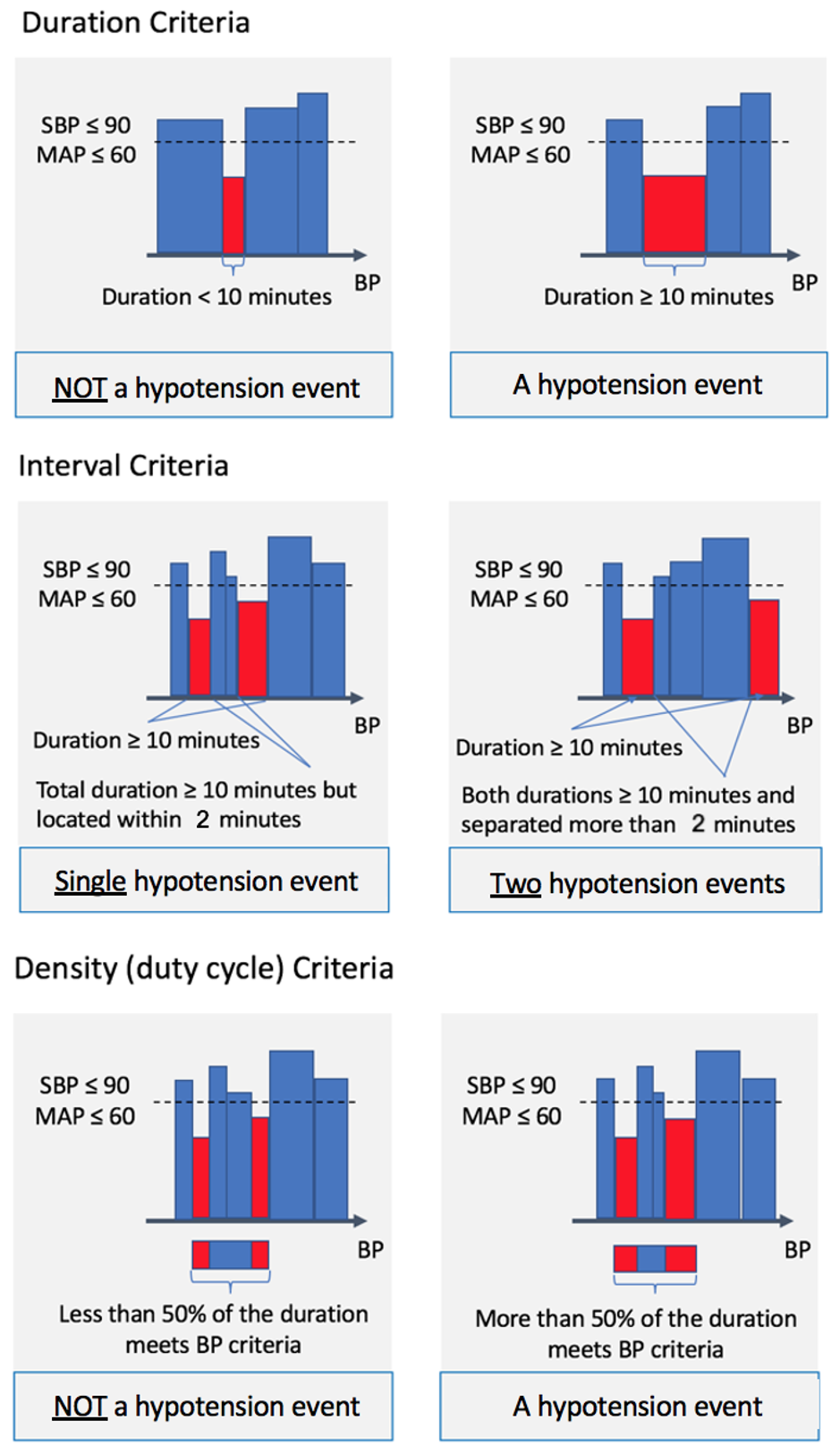

Supplement: Supplementary file 1 — Additional file 1: Figure S1. Illustration of definition for hypotension events, with duration, interval, and density criteria. [file 13054_2020_3379_MOESM1_ESM.tiff]

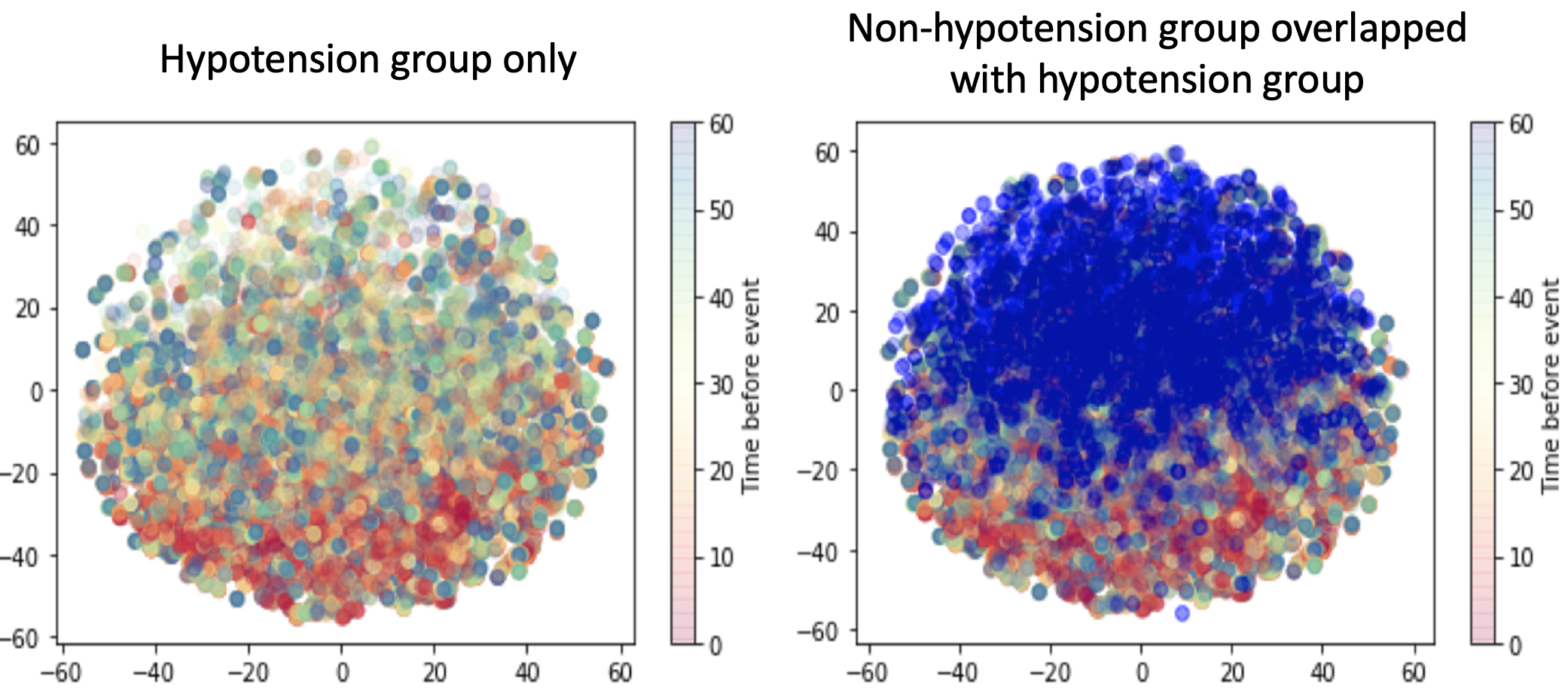

Supplement: Supplementary file 2 — Additional file 2: Figure S2. Selection of optimal time window prior to hypotension event by using a t-stochastic neighbor embedding (t-SNE) embedding. Raw vital sign data were plotted from 1 h prior to hypotension event (or 1 h prior to the average time of hypotension event, in hypotension or non-hypotension groups). The t-SNE enforces data with similar vital signs to be projected closely. With this manifold representation of data, distribution of data points for the hypotension group was shown (Left). The colors of dots represented the time horizon towards the hypotension event. The data points of the non-hypotension group (dark blue dots) overlapped onto a major portion of the hypotension group, suggesting the non-hypotension group vital sign behaviors are similar to those of the hypotension group. However, when compared to the hypotension group only (Right), the overlapping does not involve certain areas, especially red-colored dots, representing hypotension subjects at approximately 15 min before the hypotensive event. This suggests that the vital sign patterns are probabilistically different between the two groups about 15 min prior to the event. [file 13054_2020_3379_MOESM2_ESM.tiff]

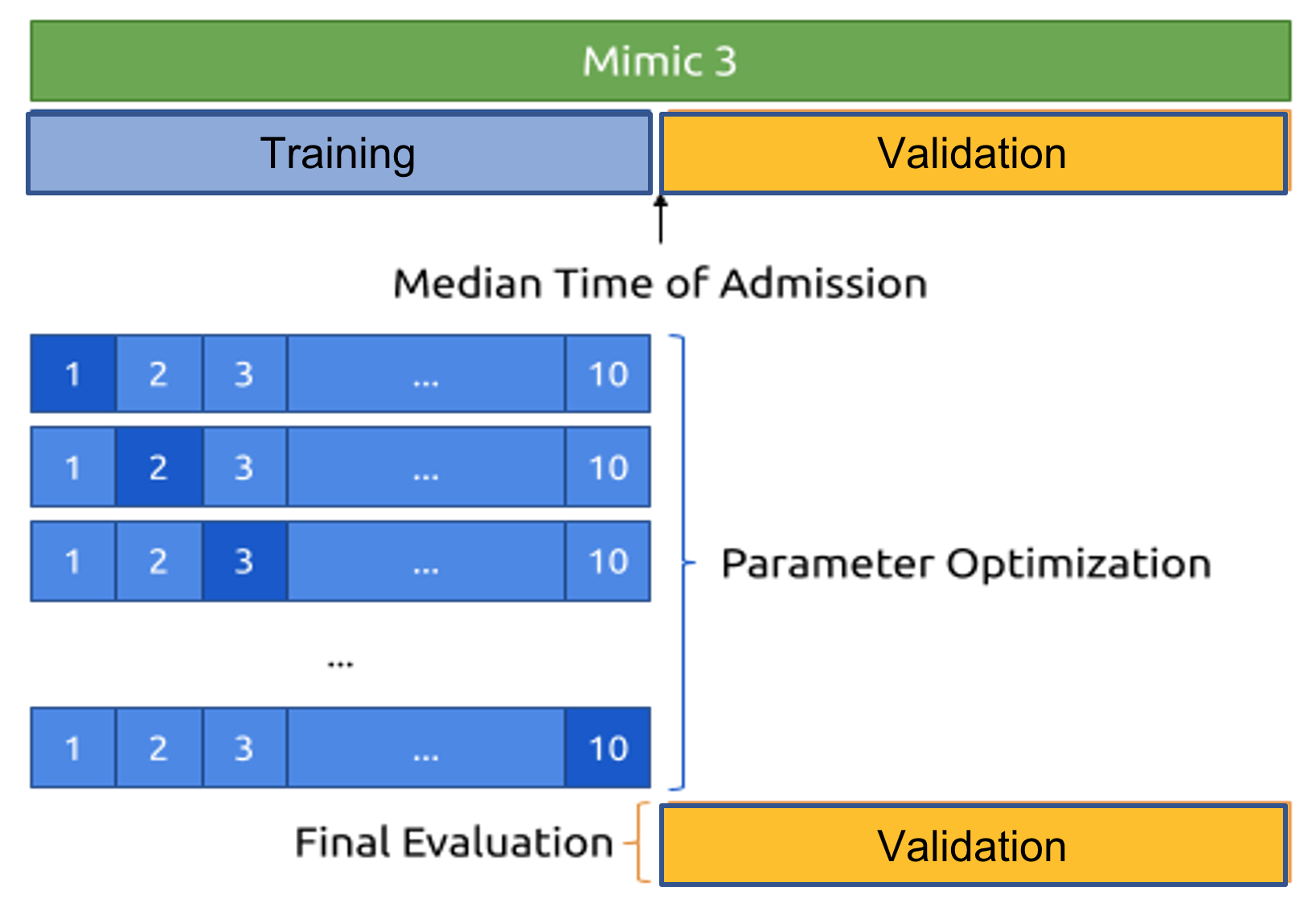

Supplement: Supplementary file 3 — Additional file 3: Figure S3. Splitting the data into training and validation subset, according to the median time of ICU admission. The blue part (Left) was used to complete the hypotension prediction model with training followed by 10-fold cross validation methods. Then the pre-separated yellow part (Right) was used to validate the model afterwards. [file 13054_2020_3379_MOESM3_ESM.tiff]

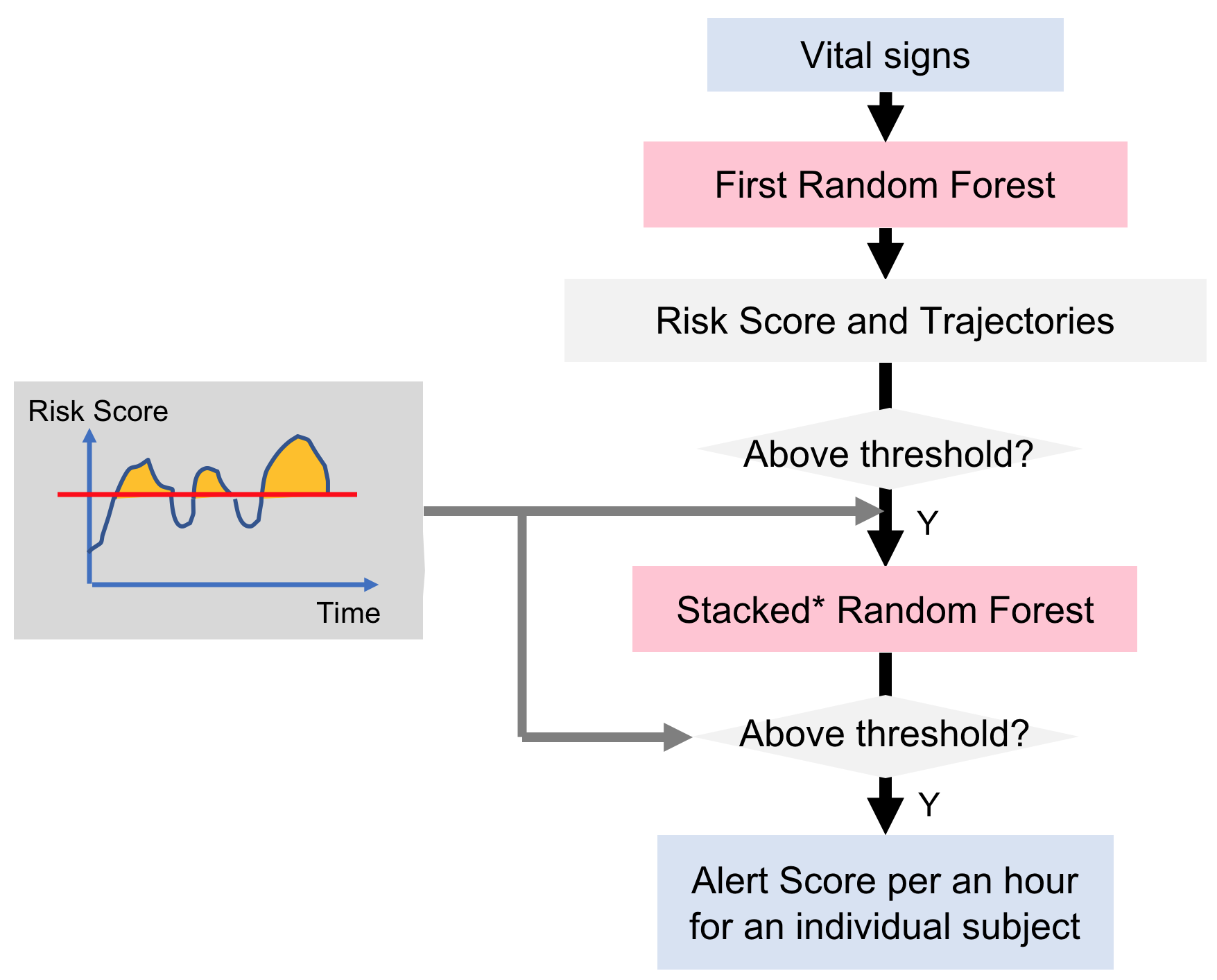

Supplement: Supplementary file 5 — Additional file 5: Figure S4. A flowchart of stacked model (two-step approach) to derive alert scores. The first step model is used a random forest regressor trained on the input vital sign features to predict risk scores. The second step model is another random forest with expanded feature set to decrease false alerts. The additional features include time since admission, average, minimum, maximum, and standard deviation of risk scores obtained from the first model, over the last 5, 10, and 30 min prior to the current time. Both random forest models were trained and tested with tenfold cross validation. [file 13054_2020_3379_MOESM5_ESM.tiff]

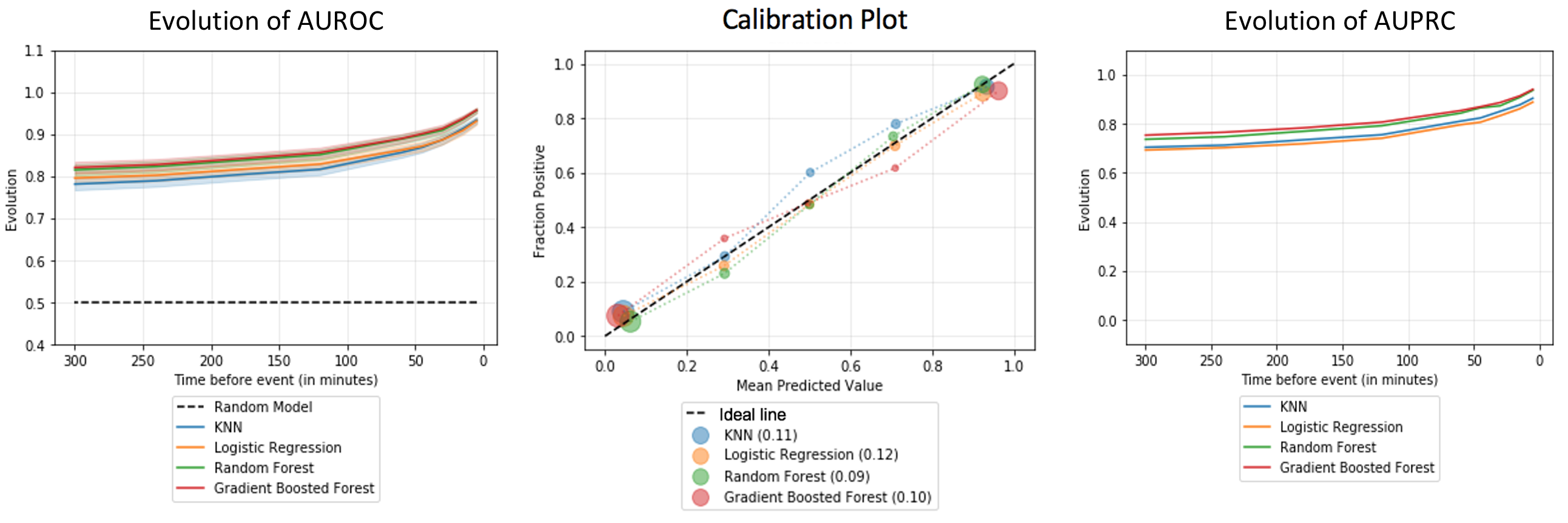

Supplement: Supplementary file 8 — Additional file 8: Figure S5. Performance evaluation for various supervised machine learning algorithms on the training cohort, with the evolution of area under the receiver operating characteristics (AUROC) over time (Left), calibration plot with the Brier’s score (Center), and the evolution of the area under the precision–recall curve (AUPRC) over time (Right). Note the random forest had similar performance to other methods in terms of AUROC and AUPRC distribution, but it demonstrated superior calibration metric. [file 13054_2020_3379_MOESM8_ESM.tiff]
