# Supplementary material for: Prediction of hypotension events with physiologic vital sign signatures in the intensive care unit
Source: Crit Care. 2020 Nov 25;24:661. doi: 10.1186/s13054-020-03379-3 (PMC7687996; doi:10.1186/s13054-020-03379-3)
Supplement: Supplementary file 4 — Additional file 4: Glossary for machine learning models, model training methods, and model performance evaluation techniques used. [file 13054_2020_3379_MOESM4_ESM.docx]

Glossary

Title: Prediction of hypotension events with physiologic vital sign signatures in the intensive care unit.

**Machine learning models**

Random forest: an ensemble method combining multiple decision trees which have been trained on different subsets of data and features. For prediction, the results of all the decision trees are combined.

Logistic regression (with regularization): A statistical model which uses a logistic function to model a binary variable based on a linear combination of independent covariates. A regularization factor can be applied on the mathematical optimization of this function to limit overfitting by enforcing uniform weights over the set of covariates.

K-nearest neighbors: A non-parametric model which classifies an object as the majority classes of its neighbors.

Gradient boosted trees: A model using multiple decision trees, sequentially trained on the pseudo residuals and combined to minimize the training error. Unlike a random forest, trees are not trained independently but in a boosting fashion.

t-SNE (t-distributed stochastic neighbor embedding): A non-linear dimensionality reduction (transformation of data to a lower dimension) algorithm displaying similar objects as nearby points in a lower dimensional space by minimizing the divergence between the distribution of pairwise distances in the original space and the distribution in the projected space.

**Model training technique**

Cross validation method: A model validation technique based on a resampling process, to reduce variance, assess generalization of a model by iteratively evaluating its performance over different set of data combination. In the process, the model is trained with a subset of data, then tested using unknown set (leftover) of data.

**Model performance analysis techniques**

Receiver Operating Characteristic curve: A graphical representation of the binary discrimination power of a model as its output threshold is varied.

Precision Recall Curve: Graphical representation of the trade-off between precision and recall.

Calibration with Brier’s score: A method to measure the alignment between predicted probabilities and observed probability distribution. (Low Brier’s score indicates better model representation of the observed distribution of probabilities.)
