# Supplementary material for: Prediction of hypotension events with physiologic vital sign signatures in the intensive care unit
Source: Crit Care. 2020 Nov 25;24:661. doi: 10.1186/s13054-020-03379-3 (PMC7687996; doi:10.1186/s13054-020-03379-3)
Supplement: Supplementary file 6 — Additional file 6: Table S1. Demographic and clinical characteristics of training and validation cohorts. [file 13054_2020_3379_MOESM6_ESM.docx]

**Table S1.**

Demographic and clinical characteristics of training and validation cohorts.

|  | Training | Validation | *Significance (p-value) |
| --- | --- | --- | --- |
| Number of stays | 1929 | 1930 |  |
| Age (years) | 62.58 (15.39) | 63.65 (15.03) | **0.043** |
| Gender (female, %) | 40.28 | 43.26 | 0.065 |
| Length of stay | 7.65 (10.33) | 8.05 (9.89) | **0.0063** |
| First ICU units (%)  Medical ICU  Surgical ICU  Cardiac ICU  Others | 9.19  37.37  11.95  41.49 | 9.94  35.31  11.02  43.73 | 0.47 |
| In-hospital Mortality (%) | 13.48 | 14.46 | 0.41 |

* Bold values are significant after Benjamini-Hochberg correction.
