# Supplementary material for: Prediction of hypotension events with physiologic vital sign signatures in the intensive care unit
Source: Crit Care. 2020 Nov 25;24:661. doi: 10.1186/s13054-020-03379-3 (PMC7687996; doi:10.1186/s13054-020-03379-3)
Supplement: Supplementary file 7 — Additional file 7: Table S2. Demographic and clinical characteristics of the training and validation cohort for Hypotension group and non-hypotension group across all subjects. [file 13054_2020_3379_MOESM7_ESM.docx]

**Table S2.**

Demographic and clinical characteristics of the training and validation cohort for Hypotension group and non-hypotension group across all subjects.

|  | Hypotension group | Non-hypotension group | *Significance (p-value) |
| --- | --- | --- | --- |
| Number of stays | 1580 | 2279 |  |
| Age (years) | 66.43 (13.96) | 60.81 (15.62) | **< 0.001** |
| Gender (female, %) | 43.16 | 40.81 | 0.15 |
| Length of stay | 10.99 (12.77) | 5.68 (6.98) | **< 0.001** |
| First ICU units (%)  Medical ICU  Surgical ICU  Cardiac ICU  Others | 15.91  26.45  19.50  38.14 | 5.72  42.32  6.63  45.33 | **<0.001** |
| In-hospital Mortality (%) | 34.11 | 0 ^+^ |  |

+ Dead non-hypotension subjects have been removed

* Bold values are significant after Benjamini-Hochberg correction.
